# C-to-U RNA deamination is the driving force accelerating SARS-CoV-2 evolution

**DOI:** 10.26508/lsa.202201688

**Published:** 2022-11-08

**Authors:** Yan Li, Fanghua Hou, Meili Zhou, Xiaoping Yang, Bin Yin, Wenqing Jiang, Huiqing Xu

**Affiliations:** 1 Cardiovasology Department I, Qingdao Center Hospital, Qingdao, China; 2 Emergency Department, Qingdao Center Hospital, Qingdao, China; 3 Department of Respiratory Diseases, Qingdao Haici Hospital, Qingdao, China; 4 Department of Pathology, Qingdao Haici Hospital, Qingdao, China

## Abstract

Understanding the molecular mechanism underlying the rampant mutation of SARS-CoV-2 would help us control the COVID-19 pandemic. The APOBEC-mediated C-to-U deamination is a major mutation type in the SARS-CoV-2 genome. However, it is unclear whether the novel mutation rate *u* is higher for C-to-U than for other mutation types, and what the detailed driving force is. By analyzing the time course SARS-CoV-2 global population data, we found that C-to-U has the highest novel mutation rate *u* among all mutation types and that this *u* is still increasing with time (d*u*/d*t* > 0). Novel C-to-U events, rather than other mutation types, have a preference over particular genomic regions. A less local RNA structure is correlated with a high novel C-to-U mutation rate. A cascade model nicely explains the d*u*/d*t* > 0 for C-to-U deamination. In SARS-CoV-2, the RNA structure serves as the molecular basis of the extremely high and continuously accelerating C-to-U deamination rate. This mechanism is the driving force of the mutation, adaptation, and evolution of SARS-CoV-2. Our findings help us understand the dynamic evolution of the virus mutation rate.

## Abstract (precis)

The authors propose that the host-mediated C-to-U RNA editing event, which is also affected by the RNA structure, is the driving force that accelerates SARS-CoV-2 mutation and evolution.

## Introduction

The endless mutations in SARS-CoV-2 sequences largely complicate our control of the pandemic. Understanding the mutational bias in SARS-CoV-2 together with its driving force is helpful for scientists to predict the trajectory of virus evolution. Thus, finding out the molecular basis underlying the mutation and fast evolution of SARS-CoV-2 would not only contribute to controlling this pandemic, but also let us better comprehend the origin and evolution of SARS-CoV-2 (Yu et al, 2021).

Among the various mutation sites in SARS-CoV-2, many literature studies have independently reported that the APOBEC-mediated C-to-U RNA deamination largely contributes to the mutation profile of SARS-CoV-2 (Simmonds, 2020; Li et al, 2020c; Simmonds & Ansari, 2021; Liu et al, 2022). That means a large proportion of the viral mutations came from C-to-U deamination by hosts (Chester et al, 2004; Blanc et al, 2019; Soleymanjahi et al, 2021). This is not surprising as APOBECs are well known for their virus-restriction roles (Mehta & Driscoll, 2002; Chen et al, 2010; Harris & Dudley, 2015). An important implication is that given the rampant C-to-U deamination on viral RNAs, the distribution of the numbers of the 12 types of nucleotide substitutions (mutations) in the SARS-CoV-2 genome should be asymmetric, where C>U mutation (representing the C-to-U deamination) should show a dominant peak in the mutation profile (Simmonds, 2020; Li et al, 2020b; Liu et al, 2022). The contribution of the other 11 non-C>U mutation types to SARS-CoV-2 evolution is minor. Although an early study by Di Giorgio et al (2020) claimed that a symmetric SNP-like mutation profile was detected in the SARS-CoV-2 transcriptome (Di Giorgio et al, 2020), the reliability of this study was soon challenged by many other scientists (Picardi et al, 2021; Cai et al, 2022; Song et al, 2022; Wei, 2022; Zong et al, 2022). Therefore, the C-to-U deamination is still believed to be the most abundant mutation type in SARS-CoV-2 and is likely to be the major force that drives virus evolution (Zhang et al, 2021). Investigating the C-to-U deamination in SARS-CoV-2 is a promising aspect for studying the driving force underneath SARS-CoV-2 mutation and evolution and helps us predict the trajectory of the ongoing pandemic.

However, several key points regarding the C-to-U deamination in SARS-CoV-2 remain unanswered. Although the observed C-to-U mutations obviously outnumber other mutation types (Simmonds, 2020; Liu et al, 2022), it is unclear (1) whether the novel mutation rate *u* (mu) of C-to-U is higher than the *u* of other mutation types, (2) whether the C-to-U mutation rate itself is increasing or decreasing with time, and (3) what is the possible molecular mechanism underlying the prevalent C-to-U deamination in SARS-CoV-2 RNA. Most mutational studies on SARS-CoV-2 only focused on the “static” profile of mutations, which is a snapshot of mutations at a particular time point (TP) (Simmonds, 2020; Li et al, 2020b; Liu et al, 2022). Notably, it needs to be clarified that (1) the observed number of mutations (*N*) at a snapshot does not fully represent the novel mutation rate (*u*), because, by definition, *N* is the accumulation of *u* during a long period; then, looking at the relative mutation rate *u*’ of each mutation type is also informative; (2) moreover, novel mutation rate *u* itself may change with time (*t*) so that looking at d*u*/d*t* (or d*u*’/d*t*) also provides us valuable information on the molecular mechanism governing the up and down of novel mutation rate *u*. Therefore, the three currently unknown questions listed by us (*u*, d*u*/d*t*, and mechanism) need to be answered.

**Figure 1.**
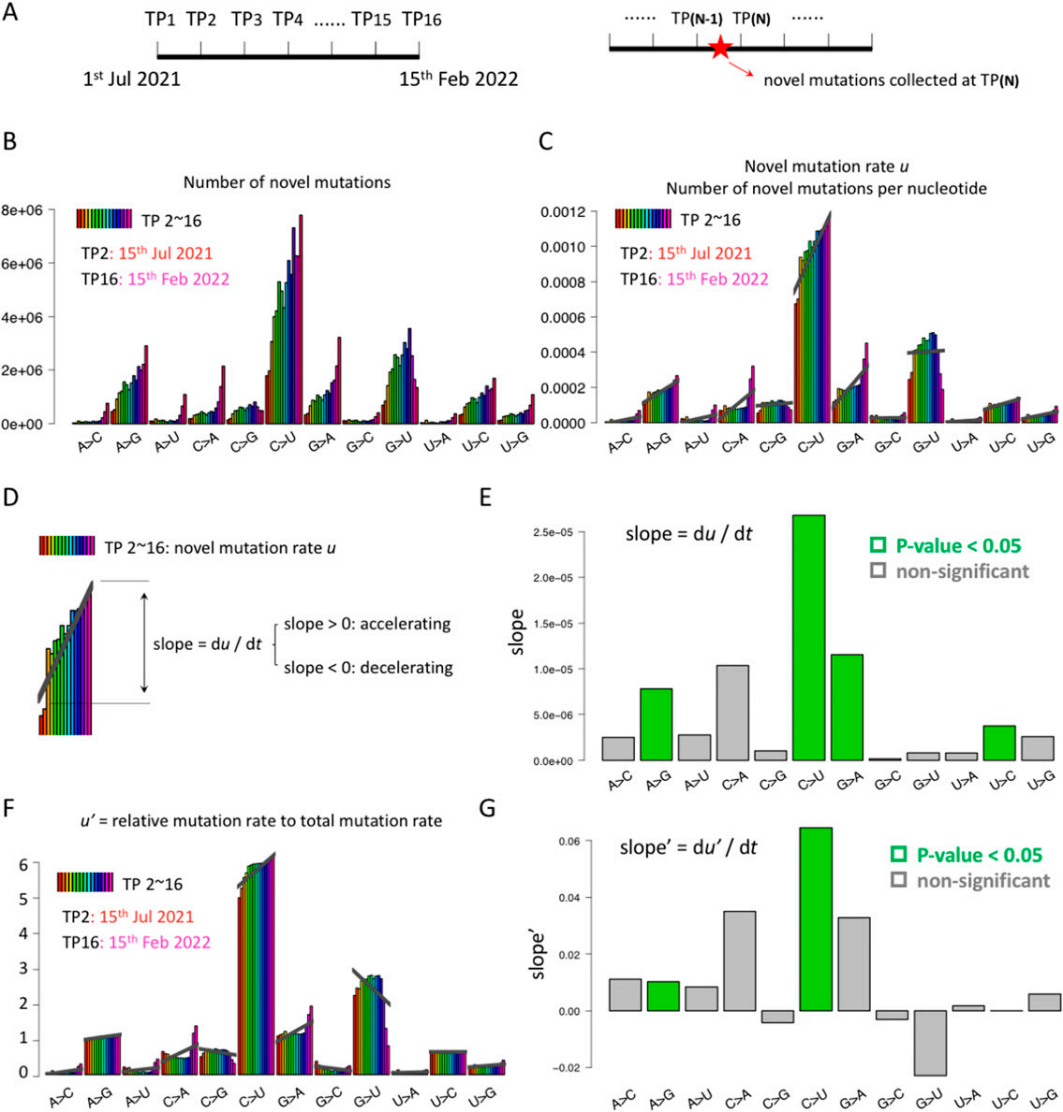
Definition and basic statistics of novel mutations at each time point. **(A)** Novel mutation events were collected within each 15-d period from July 1, 2021, to February 15, 2022. TP means time point. There are 16 TPs across this 8-mo time. In this plot, TP2 means the data collected at TP2, including the novel SARS-CoV-2 sequences produced from TP1 to TP2 (15 d). **(B)** Numbers of novel mutation events. The numbers are expected to increase with time because the number of sequenced SARS-CoV-2 sequences (within every 15 d) is increasing. **(C)** Novel mutation rate *u* is the number of novel mutation events per nucleotide. This rate is normalized by the numbers of nucleotides within the sequenced SARS-CoV-2 sequences so that the sequencing bias is canceled. The slope of each mutation type in this plot (thick gray lines) would reflect the increase or decrease in the intrinsic mutation rate for each mutation type. C-to-U mutation (C>U), representing the C-to-U RNA deamination, is the most abundant mutation type, and its mutation rate is still increasing. **(D)** Slope = d*u*/d*t*. The slope is the change of novel mutation rate *u* with time. Slope > 0 means *u* is accelerating with time. **(E)** Slope of 12 mutation types. C>U mutation has the highest slope, suggesting that the novel C>U mutation rate *u* is still increasing with time. Green bars indicate that the slope is significantly different from 0. **(C, F)** Barplots are similar to those in (C) but demonstrate the relative mutation rate *u*’. **(G)** Slope’ = d*u*’/d*t*. The green bars indicate that the slope is significantly different from 0.

In this study, we aim to address these issues. Using the time course SARS-CoV-2 population data produced by previous literature studies (Shu & McCauley, 2017; Zhu et al, 2022), we analyzed the novel mutations from July 1, 2021, to February 15, 2022 (Fig 1A). The previous study divided the 8-mo time into 16 equal periods (15 d for each period); we found that the C-to-U deamination (C>U mutation) always bears the highest novel mutation rate *u* in all time. Besides, the C>U mutation rate itself is increasing with time, suggesting d*u*/d*t* > 0. We then propose a “cascade model” that the RNA structure serves as the molecular basis of the high and continuously accelerating C-to-U deamination rates. Finally, we discussed that the rampant missense C-to-U deamination in the RDRP gene might elevate the overall mutation rate. Our work resolved the long-term mystery behind the driving force of SARS-CoV-2 mutation and evolution.

## Results

### C-to-U RNA deamination is the dominant mutation type among novel mutations

We collected the time course mutation profile of world-wide SARS-CoV-2 sequences from a previous study (Zhu et al, 2022), the data of which were originally produced using the public SARS-CoV-2 data from GISAID (Shu & McCauley, 2017). Briefly speaking, as described by the original study, the world-wide SARS-CoV-2 sequences were collected at 16 TPs equally distributed from July 1, 2021, to February 15, 2022 (Fig 1A). The period between two adjacent TPs was 15 d. At the last TP of their collection (TP16, February 15, 2022), there were already 6.72-million SARS-CoV-2 sequences available (Zhu et al, 2022). We downloaded the mutation profile provided by the original study. Note that the original study focused on the total mutations at a particular TP and did not perform analyses on novel mutations within each time interval (Zhu et al, 2022). Therefore, in this study, we defined novel mutations at each TP from the time course data. For example, novel mutations at TP2 mean the mutations emerging in the SARS-CoV-2 sequences collected from TP1 to TP2 (the 15-d period); that is, novel mutations at TP_(N)_ (where N = 2∼16) mean the mutations in the sequences collected from TP_(N-1)_ to TP_(N)_ (Fig 1A).

We first counted the number of novel mutations at each TP, with different mutation types counted separately (Fig 1B). We found that (1) the numbers of novel mutation events are increasing with time and (2) the C-to-U mutation is the dominant mutation type. Both patterns are understandable. As the world-wide SARS-CoV-2 sequences were produced at an increasing rate (from July 2021 to February 2022), the number of novel sequences collected at each TP is increasing with time, leading to our observation that TP_(N)_ always has more novel mutation events detected compared with TP_(N-1)_ (Fig 1B). Then, for the dominant C-to-U mutation, it is well known that the major mutation source of SARS-CoV-2 comes from C-to-U RNA deamination by hosts (Simmonds, 2020; Li et al, 2020c; Simmonds & Ansari, 2021; Liu et al, 2022); then, it is naturally expected to see more C>U mutations than other mutation types. Again, note that what we see here is the number of novel mutations emerged within a short period (15 d) rather than the total number of observed mutations *N* at a snapshot (where this *N* is understood as the accumulation of mutations for a long time).

So far, our observation first agrees with a known fact that the C>U mutation (representing the C-to-U RNA deamination) is the most abundant mutation type among all mutations. Then, we found something new that even when we only focused on the novel mutations within a short period, the C-to-U RNA deamination is still the major source contributing to the total mutation profile of SARS-CoV-2. Next, we realize that the number of sequenced SARS-CoV-2 sequences (per time unit) is increasing during the past 2 yr, so we need to cancel this bias and calculate the genuine novel mutation rate *u* and the relative mutation rate *u*’.

### Novel mutation rate *u* of C-to-U deamination is increasing with time: d*u*/d*t* > 0

Our explanation of the terminologies (e.g., *N*, *u*, and d*u*/d*t*) has clarified that the number of observed mutation events *N* does not equal the novel mutation rate *u*. To accurately estimate novel mutation rate *u*, we need to calculate the number of novel mutation events per time unit per nucleotide. The time unit is already controlled because our strategy is to count the novel mutations per 15-d period. Then, we should normalize the number of novel mutation events by the number of nucleotides within all the newly sequenced SARS-CoV-2 sequences within each 15-d period.

We display the novel mutation rate *u* for each mutation type at each TP (Fig 1C). Again, *u* at TP_(N)_ (N = 2∼16) means the novel mutation rate *u* in the sequences collected from TP_(N-1)_ to TP_(N)_. Here, we observed that (1) C>U mutation (C-to-U deamination) still has the highest novel mutation rate *u*, denoted as *u*_(**C>U**)_, and (2) *u*_(**C>U**)_ is increasing with time, suggesting d*u*/d*t* > 0 (Fig 1C and D) and that the increasing rate (slope = d*u*/d*t*) is obviously higher for C>U sites compared with other mutation types (Fig 1D and E). Importantly, by dividing the number of novel mutation events by the total nucleotides in the newly produced sequences, we already canceled the bias caused by the increasing number of sequenced SARS-CoV-2 sequences so that the observations here (Fig 1C–E) are unlikely to be caused by any technical artifacts. Our results would reflect the genuine intrinsic novel mutation rates.

However, even if the global mutation rate is increasing because of currently unknown reasons (maybe *trans* factors), we are still curious about the dynamics of the relative mutation rates *u*’ of these 12 types of mutations. To calculate the relative mutation rate at each TP, we normalized each *u* by the overall total mutation rate within the 15-d period to obtain *u*’ (Fig 1F). Then, even if (theoretically) the absolute mutation rates *u* of all types of mutations could increase over the 16 TPs, the relative mutation rates *u*’ of the 12 types should simultaneously have the increasing ones and decreasing ones because the rates have been normalized by the total mutation rate (Fig 1F and G). Not surprisingly, the C>U mutation rate *u*’ is still the highest among all types (Fig 1G), suggesting that our conclusion of accelerated C>U mutation is technically robust. In the following sections, we will use both absolute and relative mutation rates to investigate the evolution of C>U mutations.

Next, there are three questions to be answered: (1) Why the novel mutation rate *u* (or *u*’) is the highest for C>U mutations (C-to-U RNA deamination) among all mutation types? (2) Why the novel C>U mutation rate *u* (or *u*’) is still increasing (d*u*/d*t* > 0)? And (3) what is the potential molecular mechanism?

### Novel mutation rate *u* of C>U mutation, rather than other mutation types, is highly correlated at different TPs

**Figure 2.**
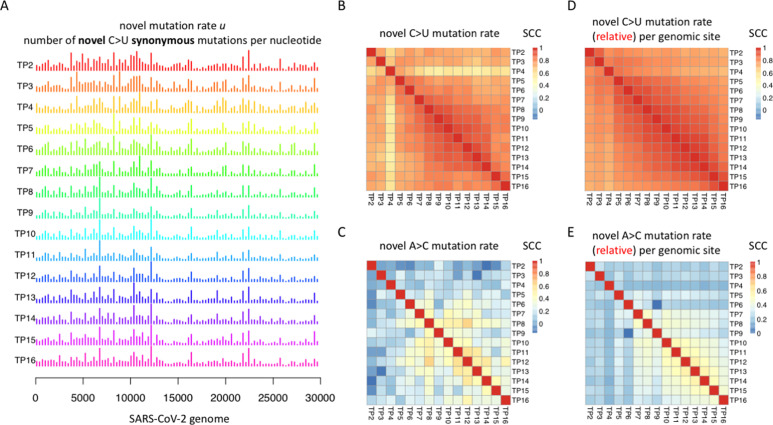
Novel mutation rate *u* of C-to-U mutations is highly correlated among each 15-d period. In this plot, TP2 means the data collected at TP2, including the novel SARS-CoV-2 sequences produced from TP1 to TP2 (15 d). **(A)** Genome-wide novel C>U mutation rates *u* of each 15-d period. The 30-Kb SARS-CoV-2 genome was divided into 100 equal segments. The median mutation rates on synonymous C>U sites within each bin were calculated. **(B)** Pairwise Spearman’s correlation coefficient (SCC) between the genome-wide novel mutation rates of each 15-d period. The synonymous C>U mutation sites were used. **(C)** Pairwise SCC between the genome-wide novel mutation rates of each 15-d period. The synonymous A>C mutation sites were used as a control. **(D)** Pairwise SCC of the per site relative mutation rate *u*’ of different time points. Synonymous C>U mutations were used. **(E)** Pairwise SCC of the per site relative mutation rate *u*’ of different time points. Synonymous A>C mutations were used as a control.

To understand the detailed evolutionary dynamics of C>U mutation (C-to-U RNA deamination), we should not only calculate the novel mutation rate *u* for the whole genome at a time (Fig 1) but also look at whether *u* varies across different genomic regions. We divided the 30-Kb SARS-CoV-2 genome into 100 equal segments (300 bp for each segment) and calculated the novel mutation rate *u* within each segment (Fig 2A). Be reminded that *u* at TP_(N)_ (N = 2∼16) represents the novel mutation rate *u* within the time frame from TP_(N-1)_ to TP_(N)_. Here, to be more stringent, we only used synonymous mutations. As we know, a mutation in the coding region might or might not change the amino acid sequence (the mutations are termed missense and synonymous mutations, respectively). Obviously, missense mutations have stronger effects on the fitness of the organism, whereas synonymous sites are believed to be neutral and are not affected by natural selection (Kimura, 1979; McDonald & Kreitman, 1991).

We observed that (1) different genomic regions have distinct novel mutation rates for C>U mutations (Fig 2A), with several mutational hotspots such as the region around 22.5 Kb. Interestingly, the genomic location of the spike (*S*) gene ranges from 21.6 to 25.5 Kb (Kent et al, 2002), and our result perfectly agrees with that of a previous report that the *S* gene is subjected to rampant C-to-U RNA deamination by host (Liu et al, 2022), leading to locally higher mutation accumulation in the *S* gene; and (2) however, although the novel C>U mutation rate *u* is unevenly distributed across the genome, strikingly, the *u* of different genomic regions is highly correlated at different TPs (Fig 2A). Theoretically, the novel mutations taken place at different TPs should be completely independent because the probability of seeing a new mutation at a position has nothing to do with whether this position had a mutation before. Next, we wondered whether this pattern is unique to C>U mutations or it is commonly seen in other mutation types as well.

We performed the pairwise Spearman correlation analysis among the novel mutation rate *u* of different genomic regions at different TPs. For C>U mutations, the *u* profiles across the genome are highly correlated among different TPs (Fig 2A and B). The pairwise Spearman correlation coefficients (SCCs) are higher than 0.8 (Fig 2B). In sharp contrast, for other mutation types like A>C mutations, the pairwise SCCs among different TPs are lower than 0.3 (Fig 2C). Intriguingly, the results seen in A>C mutations are more understandable to us because we think the novel mutation is random so that the mutation rate *u* should be mutually independent at different TPs. For the highly correlated *u* profile for C>U mutations (Fig 2B), we need to find a molecular mechanism to explain why the novel C>U mutations (C-to-U RNA deamination events) have a strong preference over particular genomic regions all the time. Before looking for the mechanism, we should ensure that our observation is not an artifact caused by technical issues like the algorithm or the positional bias of the virus genome.

We tested whether the correlation patterns are robust using the relative mutation rate *u*’. For each type of mutation at a particular TP, we calculated the relative mutation rate *u*’ per site (rather than per 300-bp region) and compared the correlation of *u*’ between different TPs. To our expectation, the *u*’ of the C>U mutation was highly correlated across different TPs (Fig 2D), whereas the *u*’ of the A>C mutation (as a control) did not show a strong correlation between TPs (Fig 2E). This again hints that the prevalent C-to-U deamination events are not randomly taking place like other mutations. Instead, there should be a molecular mechanism that ensures the high and stable occurrence rate of C-to-U deamination.

Regarding the high mutation rate on the spike (*S*) gene observed by us (Fig 2A), although the terminology “mutation hotspot” has already been mentioned by SARS-CoV-2 studies (Pachetti et al, 2020) (which seems to explain the mutational bias), this term usually refers to the unevenly distributed number of mutations (*N*) seen from a snapshot rather than referring to the strictly defined novel mutation rate *u* mentioned by us. Again, observing more mutations (total number: *N*) accumulated in a region does not necessarily prove higher novel mutation rates *u* in that region. Therefore, the “mutation rate (*u*) hotspot” theory (concept) has not been studied yet. Moreover, another aforementioned question remains unknown: Why is d*u*/d*t* > 0 for C>U mutations? In other words, why is C>U mutation rate *u* consistently increasing? Next, we aim to address these issues. Notably, although d*u*/d*t* > 0 is most evident for C>U mutation, the overall d*u*/d*t* is greater (or slightly greater) than 0 for many other mutation types. We will discuss the possible causal factors in the last section of Results.

### RNA structure accounts for the biased local C>U novel mutation rate *u*

We set out to investigate (1) why the novel C>U mutations (C-to-U RNA deamination events) have a strong preference over particular genomic regions all the time? And (2) why is d*u*/d*t* > 0 especially for C>U mutations?

**Figure 3.**
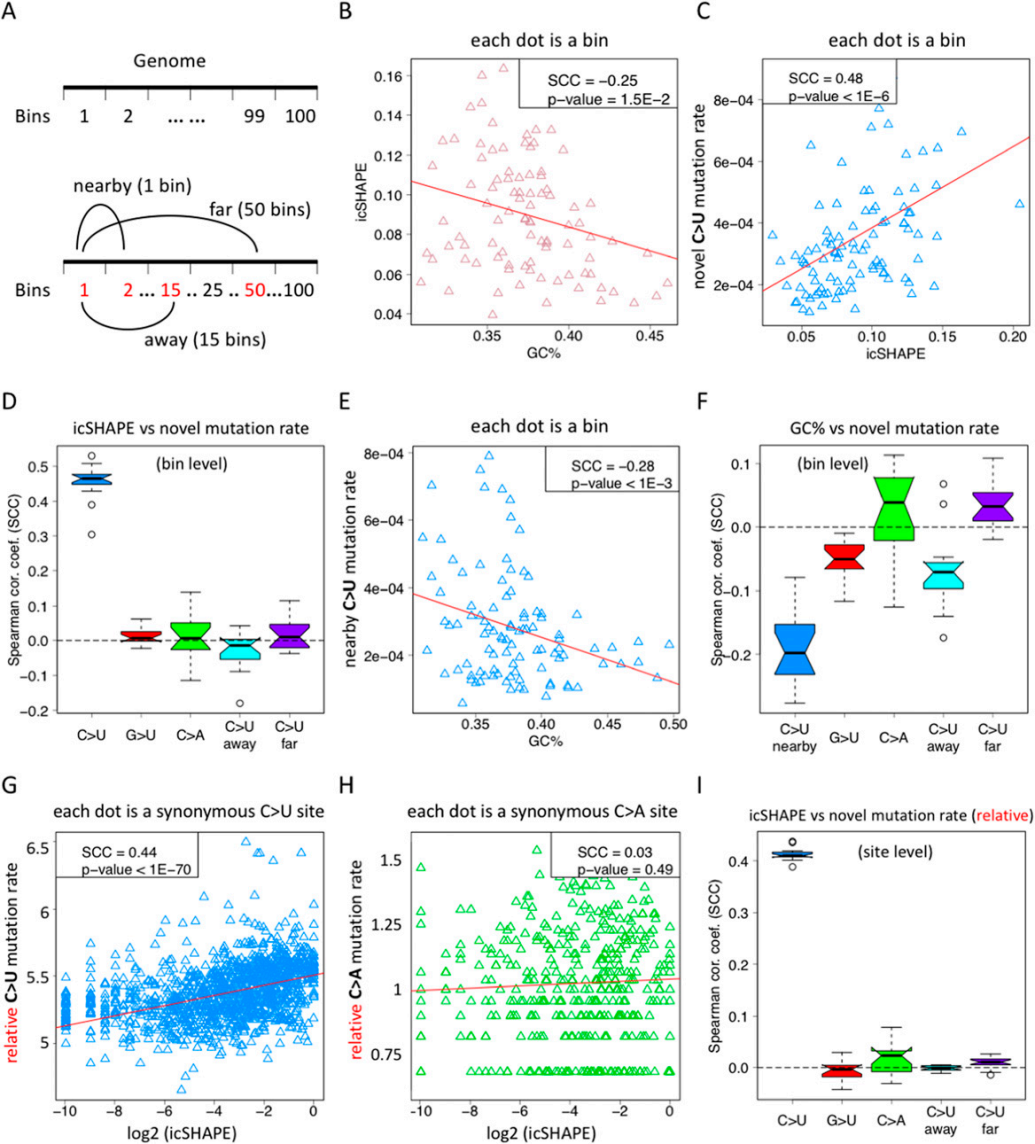
RNA structure is the basis of C-to-U deamination and explains the intrinsically high C>U mutation rate. **(A)** Definition of some terminologies. The 30-Kb SARS-CoV-2 genome was divided into 100 equal segments (bins). Nearby bins are defined as consecutive bins like bin_(N-1)_ and bin_(N)_. Away is defined as distance = 14 bins, like bin_1_ and bin_15_, or bin_(N-14)_ and bin_(N)_. Far is defined as distance = 49 bins, like bin_1_ and bin_50_, or bin_(N-49)_ and bin_(N)_. **(B)** GC contents of these 100 bins are negatively correlated with the icSHAPE score of bins. A lower icSHAPE score means a stronger RNA secondary structure. SCC, Spearman’s correlation coefficient. **(C)** Novel C>U mutation rate within each bin is positively correlated with the icSHAPE score of bins. The mutation rate in (C) was retrieved at TP2. **(C, D)** Each of the 16 TPs has an SCC value like (C) so that the 16 SCC values are displayed as box-and-whisker plots. The icSHAPE score is correlated with C>U novel mutation rate within each bin, but is not correlated with the G>U mutation rate, the C>A mutation rate, or the C>U mutation rates in farther bins. **(E)** GC content of each bin is negatively correlated with the novel C>U mutation rate in the nearby bin. The mutation rate in **(E)** was retrieved at TP2. We could not directly compare the GC content versus the C>U mutation rate within the same bin because the C>U rate is largely affected by cytidine content (and thus introducing a bias). **(E, F)** Each of the 16 TPs has an SCC value like (E) so that the 16 SCC values are displayed as box-and-whisker plots. The GC content is correlated with the C>U novel mutation rate in the nearby bin, but is not correlated with the G>U mutation rate, the C>A mutation rate, or the C>U mutation rates in farther bins. **(G)** Relative C>U mutation rate *u*’ at each single site is positively correlated with the icSHAPE score of the corresponding genomic site. The mutations at TP2 were used. **(H)** Relative C>A mutation rate *u*’ at each single site is not correlated with the icSHAPE score of the corresponding genomic site. The mutations at TP2 were used. **(G, H, I)** Each of the 16 TPs has an SCC value like (G) and (H). The 16 SCC values are displayed as box-and-whisker plots. The icSHAPE score is correlated with C>U novel mutation rate, but is not correlated with the G>U mutation rate, the C>A mutation rate, or the C>U mutation rates at distant sites. Here, “away” is defined as distance = 5 Kb and far is defined as distance = 15 Kb.

To answer these questions, we try to dig deeper into the SARS-CoV-2 genome. For the 100 equal bins (segments) of the 30-Kb SARS-CoV-2 genome (Fig 2A), we picked two (a pair of) bins and defined the following relationships between them (Fig 3A): “nearby” bins are defined as two consecutive bins like bin_(N-1)_ and bin_(N)_; “away” is defined as two bins with distance = 14 bins, like bin_1_ and bin_15_, or bin_(N-14)_ and bin_(N)_; and “far” is defined as two bins with distance = 49 bins, like bin_1_ and bin_50_, or bin_(N-49)_ and bin_(N)_ (Fig 3A). Then, we will examine what feature of these bins might account for the biased C>U novel mutation rate *u*.

It was reported that the RNA structure affects the efficiency of C-to-U deamination: the human C-to-U deamination enzyme APOBEC (and the C-to-U enzyme in other eukaryotes) prefers single-stranded RNAs (ssRNAs) (Yin et al, 2013; Severi & Conticello, 2015; Chu & Wei, 2020; Liu et al, 2022). As commonly defined, ssRNA means less RNA structure, whereas double-stranded RNA (dsRNA) means stronger RNA structure. We interrogate whether the RNA structure is a major determinant of the biased distribution of the C-to-U deamination rate. Notably, local RNA structures along the SARS-CoV-2 genome have been measured by in vivo experiments (Sun et al, 2021) and in silico estimations (Zhu et al, 2022). The single-base resolution in vivo icSHAPE score (Sun et al, 2021) is negatively correlated with the RNA structure, which means a higher icSHAPE score of a region suggests that this region is more likely to be ssRNA. The in silico estimation of the RNA structure generally assumes that a region with higher GC content is more likely to form an RNA secondary structure (dsRNA) (Zhu et al, 2022). Indeed, we found that among the 100 genomic bins of SARS-CoV-2, the icSHAPE score is significantly negatively correlated with the GC content (Fig 3B). It agrees with a known notion that the higher GC content leads to a stronger RNA structure (lower icSHAPE score).

We further observed that the icSHAPE score of each local region is significantly positively correlated with the novel C>U mutation rate (Fig 3C). This is strong evidence that the local regions with a higher icSHAPE score (more likely to be ssRNA) have higher C-to-U deamination efficiency, which is in accordance with previous knowledge on the structural basis of the APOBEC-mediated C-to-U deamination (Liu et al, 2022). Moreover, the novel C>U mutation rate shown here (Fig 3C) was the *u* observed at TP2. There are other *u* values at different TPs. Strikingly, the C>U *u* values observed from TP2 from TP16 all showed a positive correlation with a icSHAPE score (Fig 3D), supporting that the C-to-U RNA deamination prefers ssRNA regions. In contrast, the local icSHAPE score is not correlated with the local G>U or C>A mutation rates (Fig 3D). Furthermore, the local RNA structure does not affect the C>U mutation rates away or far away from the local bin (Fig 3A and D).

These results indicate that the RNA structure is a major determinant of the local C-to-U deamination rate. ssRNA regions have higher local C-to-U efficiency because of the preference for APOBEC. However, the RNA structure does not affect the local mutation rates of other mutation types (because the other types of mutations are mainly introduced by replication errors) and does not affect the C-to-U rates of far-away regions either.

The RNA structure is partially determined by the GC content of local (Fig 3B) or nearby regions (Hofacker, 2003). One may predict that the regions with lower GC content are more likely to be ssRNAs so that they should have higher local C-to-U deamination rates. However, this correlation test could not be properly performed because of the fact that cytidine is the source of the C-to-U mutation so that the regions with lower GC content definitely have less C-to-U events than those regions with higher GC content (and thus, the expected correlation may be reversed). Nevertheless, because the local RNA structure is also affected by the GC content of nearby regions, we could test the correlation between the local GC content versus the C-to-U deamination rate of nearby regions (Fig 3E). Amazingly, a negative correlation was found by us (Fig 3E and F), suggesting that the lower GC content of the local region may cause a less RNA structure of the nearby region and thus facilitating the C-to-U deamination events in nearby regions. Similarly, we tested the effect of the local GC content on local G>U and C>A rates, and the C-to-U rates of away and far-away regions, and observed relatively weaker correlations (Fig 3F).

Similar to the correlation tests between the mutation rate and the icSHAPE score (Fig 3C and D), we calculated the per site relative mutation rate *u*’ and performed correlation tests between the *u*’ and the RNA structure represented by icSHAPE. Only synonymous sites were used. We found that the *u*’ of C>U sites was positively correlated with the icSHAPE score (Fig 3G), suggesting that ssRNA boots the efficiency of C-to-U deamination. In contrast, the *u*’ of the C>A mutation was not correlated with the RNA structure (Fig 3H). Moreover, when we looked at the correlation between the local RNA structure and the *u*’ of distant C>U sites, the correlation disappeared (Fig 3I). All these results prove that (1) ssRNA drives C-to-U deamination; and (2) the correlation between the C>U mutation rate and the RNA structure is not an artifact caused by the overall increasing mutation rate because no correlation was observed between the RNA structure and other mutation types.

### Cascade model explains the increasing rate of C-to-U deamination (d*u*/d*t* > 0)

**Figure 4.**
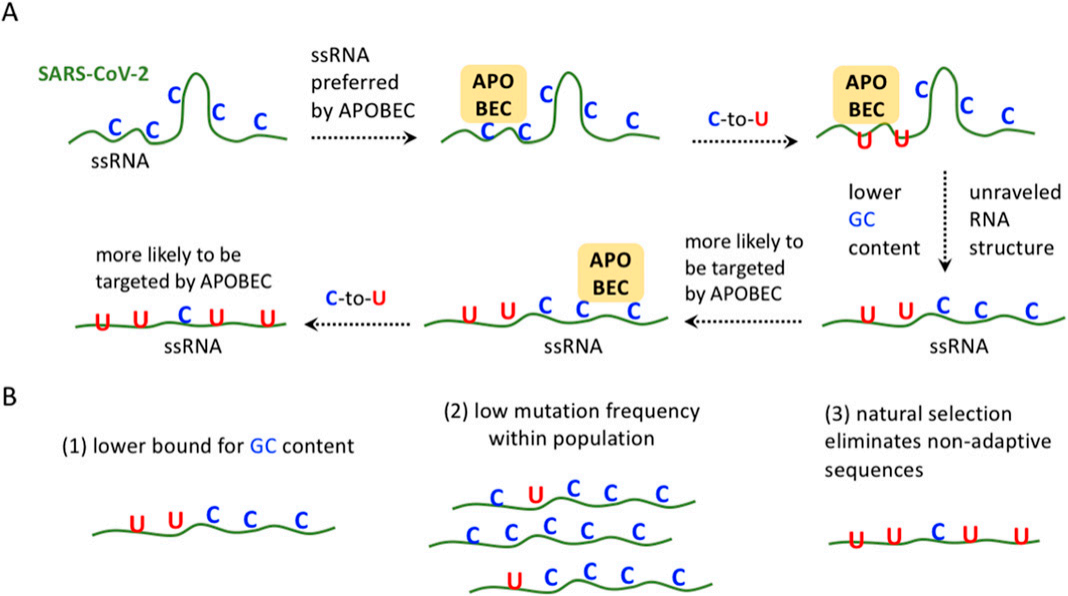
Model explaining the accelerating C-to-U deamination rate in SARS-CoV-2 and the potential limitations. **(A)** APOBEC-mediated “cascade model” (a positive feedback loop). Previous C-to-U deamination events in the SARS-CoV-2 genome would lead to a lower GC content of the local region, reducing the RNA structure of nearby regions and consequently leading to a higher probability of C-to-U deamination in nearby regions because of the preference for APOBEC. **(B)** Potential mechanisms that stop the endless C-to-U deamination: (1) there might be a lower bound for GC content; (2) for each genomic site, the deaminated SARS-CoV-2 sequences only represent a small part of the global population; and (3) natural selection will eliminate the sequences with low fitness.

We propose a “cascade model” to explain why the C-to-U deamination rate *u* is still increasing with time (d*u*/d*t* > 0). The C-to-U deamination events would lead to a lower GC content of the local region, reducing the RNA structure of local (or nearby) regions and consequently leading to a higher probability of C-to-U deamination in local (or nearby) regions because of the preference for APOBEC (Fig 4A). Then, the APOBEC-mediated C-to-U deamination further reduces the GC content and makes the genome RNA more suitable for C-to-U deamination. This “chain reaction” would cause an unstoppable cascade at a genome-wide level. More and more cytidine sites are deaminated to uridines, reducing the local GC content and the RNA structure, leading to a new “round” of C-to-U deamination events by APOBEC in local (or other) regions. Each “round” of C-to-U deamination would again reduce the genomic GC content and elevate the probability of the next “round” of C-to-U deamination (Fig 4A). The molecular process under this scenario is similar to a positive feedback loop, and this cascade might explain the continuously increasing C-to-U deamination rates (d*u*/d*t* > 0). Therefore, the RNA structure might be the driving force underlying the rampant C-to-U deamination and thus the fast evolution of SARS-CoV-2. However, there should be a mechanism to circumvent an extremely low CG content because of the rampant C-to-U deamination. We will discuss the possible mechanisms in the Discussion section (Fig 4B).

### Missense C-to-U deamination sites in RDRP may affect the global replication error rate

All our analyses above are based on synonymous sites to exclude the effect of natural selection on estimating the mutation rate. Here, we would like to briefly investigate the missense mutations in the SARS-CoV-2 genome. We have observed that the global mutation rate seems to be increasing with time although the C>U mutation is the most prominent one among all mutation types (Fig 1E). Possibly, some *trans* factors might account for the increasing total mutation rate. Intriguingly, SARS-CoV-2 encodes an RDRP (RNA-dependent RNA polymerase). If RDRP is damaged by missense mutations, then this enzyme might become error-prone and produce more replication errors, leading to the overall increasing mutation rate.

**Figure 5.**
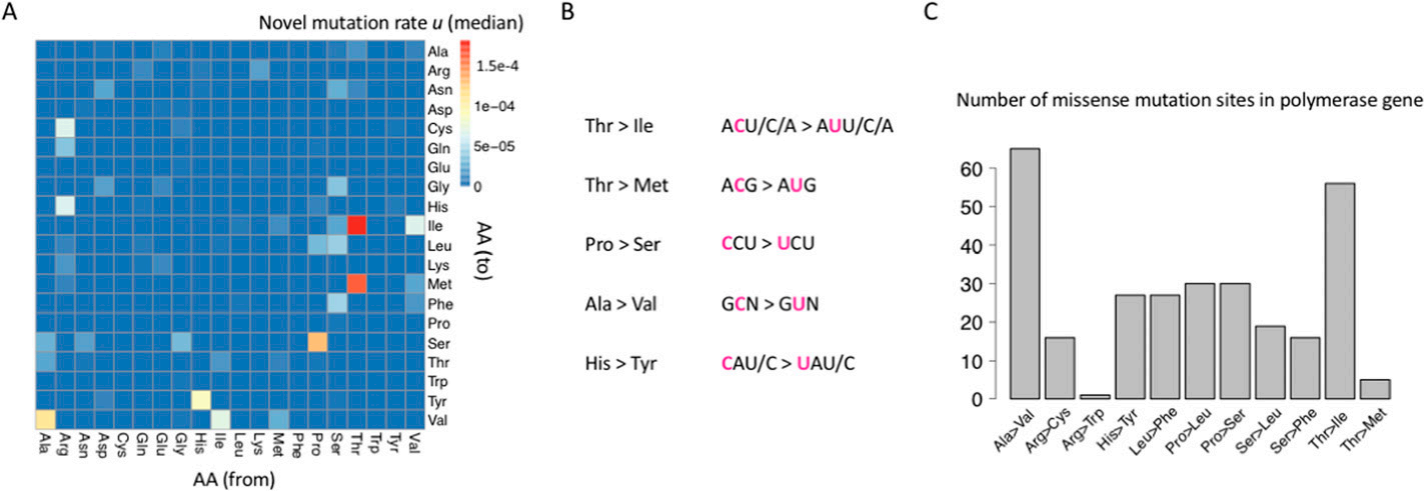
Missense mutations in the SARS-CoV-2 genome. **(A)** Novel mutation rate *u* of all missense mutations. Missense mutations cause the change of amino acid (AA). The x-axis is the encoded AA before mutation (from). The y-axis is the encoded AA after mutation (to). **(B)** AA changes and the corresponding codon changes of the most prevalent missense mutations in the SARS-CoV-2 genome. All these prevalent mutations are mediated by C>U mutations. **(C)** SARS-CoV-2 encodes an RDRP (RNA-dependent RNA polymerase). The numbers of missense mutations in the RDRP gene are shown as barplots. The most abundant mutations are also mediated by C>U.

We calculated the mutation rate for all missense mutations (Fig 5A). We selected the amino acid (AA) alterations with the highest mutation rates and checked their codon changes. We found that the top 5 AA alterations were caused by C>U mutations (Fig 5B). This suggests that the C-to-U deamination mediated by host’s APOBECs also largely contributes to the missense mutations. Expectedly, the most prevalent missense mutations in the RDRP gene (Ala>Val and Thr>Ile) are also caused by the C>U mutation (Fig 5C). Therefore, the missense C-to-U deamination could consequently interplay with a global mutation rate.

## Discussion

By parsing the time course mutation profile of the SARS-CoV-2 genome, we tracked the dynamics of novel mutations. We found that (1) the C>U novel mutation rate *u* (C-to-U deamination efficiency) is remarkably higher than the novel mutation rates of other mutation types; (2) the C>U novel mutation rate *u* is increasing with time; (3) the C>U novel mutation rate *u* has a strong preference, the *u* of different genomic regions is highly correlated at different TPs, but the other mutation types are not; (4) the RNA structure explains the biased distribution of novel C>U mutation rate *u* across the SARS-CoV-2 genome; and (5) the RNA structure may explain d*u*/d*t* > 0 for C>U mutations (the accelerating emergence rate of C-to-U deamination events).

The last explanation on d*u*/d*t* > 0 remains speculative. There are some theoretic difficulties to be resolved before finding a perfect explanation for the accelerating C-to-U deamination rate. We have proposed a APOBEC-mediated “cascade model” (or termed a positive feedback loop) that describes the unstoppable C-to-U deamination events in the SARS-CoV-2 genome (Fig 4A): previous C-to-U events lead to a lower GC content and a less RNA structure and then trigger new C-to-U events. However, here is a dilemma: the genomic cytidines are not infinite. Once all the cytidines in the SARS-CoV-2 genome are replaced with uridines, then the GC content would be very low, and the dsRNA structure would be rare and weak, and this situation is favorable for C-to-U deamination. However, no cytidines are available at that time.

To solve this dilemma, (1) we first speculate that there should be a lower bound of GC content (or cytidine content) in the SARS-CoV-2 genome (Fig 4B). This means that the C-to-U deamination rate could not accelerate forever. Despite that, we currently observed d*u*/d*t* > 0 for C>U mutations, once the genome has very low cytidine content, then the C>U rate might slow down and d*u*/d*t* will be reversed (Fig 4B). Then, (2) although currently we see d*u*/d*t* > 0 for C>U mutations, the absolute value of d*u* is still very low. This is because the mutation sites are usually found with low allele frequency < 1/1,000 within the SARS-CoV-2 population (Fig 4B). The mutated (deaminated) sequences only represent a small part of the global population. When we look at a particular site, most of the SARS-CoV-2 sequences in the world are the “wild-type” sequence (unmutated version). Therefore, the current C-to-U deamination events are still very hard to significantly reduce the genomic GC content when taking the allele frequency into account. Finally, (3) the evolution of genomic sequence is not only affected by mutation rates but also governed by natural selection. In the evolutionary arms race between humans and SARS-CoV-2, the virus has to extensively mutate and then select the most favorable sequences in order to compete with host (endogenous) RNAs (Wang et al, 2021; Zhang et al, 2022). Conceivably, once a heavily deaminated sequence (potentially with low GC content) has very low fitness, then this sequence would be purged by purifying selection (Fig 4B). A solid example is that an excessively low GC content in the SARS-CoV-2 genome is unfavorable for the translation of viral RNAs (Li et al, 2020a), and therefore, this “low-GC virus strain” might be less adaptive and be eliminated. In other words, purifying selection has maintained the GC content in the SARS-CoV-2 genome. The first two possibilities stress the molecular mechanisms, whereas the third possibility (natural selection) might be the most powerful strength that maintains a reasonable GC content of the virus.

Another confusing observation is that although the cascade model explains the d*u*/d*t* > 0 for C-to-U deamination, it does not explain why some other mutation types still have a positive d*u*/d*t* value (although lower than that of C>U) (Fig 1). In theory, apart from C-to-U deamination and the ADAR-mediated A-to-I(G) deamination (Chen et al, 2000; Palladino et al, 2000; Tian et al, 2008), the other 10 mutation types in SARS-CoV-2 mostly come from RNA replication errors (Di Giorgio et al, 2020; Cai et al, 2022) and should not be affected by genome architecture (*cis* elements). Therefore, we propose a possibility that the *trans* factors like RDRP account for the overall d*u*/d*t* > 0. When RDRP is subjected to rampant missense C>U mutations (Fig 5), it might become error-prone and produce more replication errors. We admit that there might be unnoticed bias in the definition of d*u*/d*t*, which might cause a tendency of d*u*/d*t* > 0 regardless of the mutation type. However, even if this unnoticed bias exists, C>U still bears the highest d*u*/d*t* value (Fig 1). This means that when the potential bias is removed (say, by calculating the relative mutation rate *u*’), C>U is still likely to have the highest d*u*/d*t* value, which is greater than 0. Therefore, the cascade model for C-to-U deamination remains valid.

All in all, by investigating world-wide SARS-CoV-2 sequences, we reveal that the C-to-U RNA deamination is the driving force accelerating SARS-CoV-2 evolution. Our study should be interesting to the broad SARS-CoV-2 researchers and evolutionary biologists.

## Materials and Methods

### Data collection

We downloaded the time course mutation profile of world-wide SARS-CoV-2 sequences from the supplementary data of a previous study (Zhu et al, 2022) (https://www.tandfonline.com/doi/suppl/10.1080/15476286.2022.2092351/suppl_file/krnb_a_2092351_sm0926.xlsx).

The data of that original study were produced using the public SARS-CoV-2 data from GISAID (Shu & McCauley, 2017). Briefly speaking, the world-wide SARS-CoV-2 sequences were collected at 16 TPs equally distributed from July 1, 2021, to February 15, 2022. The period between TP_(N)_ and TP_(N-1)_ was 15 d. At the last TP (TP16, February 15, 2022), there were already 6.72-million SARS-CoV-2 sequences available (Shu & McCauley, 2017; Zhu et al, 2022). We downloaded the supplementary data provided by the original study (Zhu et al, 2022). The dataset is a matrix, each row is a mutation site in the SARS-CoV-2 genome (given by genomic coordinate), and each column is the mutational allele frequency at a TP. There are totally 16 TPs. There is an additional column in the matrix providing the genomic location of the mutation, which is the information on whether this is a missense or synonymous mutation, and whether this mutation is in the coding region or untranslated region. This enables us to directly extract the synonymous mutations when we need to.

We defined novel mutations at each TP. For example, novel mutations at TP2 mean the mutations emerging in the SARS-CoV-2 sequences collected from TP1 to TP2 (the 15-d period); that is, novel mutations at TP_(N)_ (where N = 2∼16) mean the mutations in the sequences collected from TP_(N-1)_ to TP_(N)_.

To calculate the novel mutation rate *u*, we normalized the number of novel mutation events by the number of nucleotides within all the newly sequenced SARS-CoV-2 sequences within each 15-d period. This rate refers to the absolute novel mutation rate *u*.

Next, we calculated the relative mutation rate of each type of mutation (i.e., A>G, C>U). At each TP, we normalized each *u* by the overall total mutation rate within the 15-d period. Then, even if (theoretically) the absolute mutation rates of the 12 types of mutations could increase over the 16 TPs, the relative mutation rates of the 12 types should simultaneously have the increasing ones and decreasing ones because the rates have been normalized by the total mutation rate.

### Statistics and visualization

All statistical tests including correlation tests were performed using the R language. All graphic works were visualized using RStudio.

The slope was calculated using the function “lm” in R. For example, two vectors X and Y have the same length, and then, the slope of the regression line was retrieved by command line summary(lm(Y∼X)). The *P*-value of the regression was also given in the summary. *P* < 0.05 suggests that the slope is significantly different from 0.

The SCC and the corresponding *P*-values were calculated using command line cor.test(X, Y, method = “spearman”) in R. The median value of a set of numbers was calculated using the function “median” in R.

### Data access

The data were collected from a previous study (Zhu et al, 2022) and were also publicly available in GISAID (Shu & McCauley, 2017).

## Supplementary Material

Reviewer comments
